# ZIEL: Internet-Based Self-Help for Adjustment Problems: Results of a Randomized Controlled Trial

**DOI:** 10.3390/jcm8101655

**Published:** 2019-10-11

**Authors:** Christian Moser, Rahel Bachem, Thomas Berger, Andreas Maercker

**Affiliations:** 1 Clinical Psychology and Psychotherapy, University of Bern, 3012 Bern, Switzerland; thomas.berger@psy.unibe.ch; 2 Psychopathology and Clinical Intervention, University of Zürich, 8050 Zürich, Switzerland; rahel.bachem@gmail.com (R.B.); maercker@psychologie.uzh.ch (A.M.)

**Keywords:** adjustment disorder, e-mental health, self-guided intervention, disorders specifically related to stress

## Abstract

Adjustment Disorder (AjD) represents a healthcare paradox. On the one hand, it is one of the most diagnosed mental disorders worldwide. On the other hand, AjD and its possible treatment options remain a severely neglected field of research. In this context, we developed a self-guided online intervention for adjustment problems, named ZIEL, and tested its efficacy. It is based on and extends a bibliotherapeutic treatment approach for symptoms of AjD. In our study, a total of 98 individuals who had experienced a life event in the last two years, were randomly assigned to care as usual (CAU) or an online intervention group (CAU + online intervention). The primary endpoint was AjD symptom severity measured by Adjustment Disorder–New Module 20 (ADNM-20). Secondary endpoints were depressive symptoms, quality of life and other variables such as satisfaction and usability. Both the intervention and the control group improved comparably well regarding the severity of adjustment disorder symptoms post-treatment. However, participants in the intervention group showed significantly fewer depressive symptoms and a significantly higher quality of life (Cohen’s *d*: 0.89 (BDI) and −0.49 (SF-12)). The intervention was well-received by users with an above average usability rating. Overall, the results suggest that the ZIEL intervention has the promise to contribute to the treatment of AjD and reduce symptom burden by means of a scalable low-barrier approach.

## 1. Introduction

“It’s really wonderful how much resilience there is in human nature,” famous Dracula novelist Stoker stated at the end of the 19th century, against the background of his own adverse experiences [1]

However, there are events in the lives of people that can exceed their psychological resilience. Adjustment Disorder (AjD) marks the transition between normal stress response and impairments with clinically relevant severity or duration [2]. Even if the specific nature of this shift is debated, it does exist [3,4]. What is remarkable, however, is the extent to which the state of clinical practice diverges from the research efforts on AjD. On the one hand, it is one of the most diagnosed mental disorders worldwide [5]. On the other hand, research on AjD and its possible treatment remain a severely neglected field of study [6].

One reason for the gap between practice and research is the lack of diagnostic reliability and validity of AjD, in its ICD-10 and DSM-5 sub-threshold conceptualization [7,8,9]. However, the evidence base for a full-threshold understanding has been increasing. Amongst others, this includes an equal symptom-severity to e.g., major depression, a lowered quality of life and comparably earlier execution of suicidal ideations [10,11,12,13]. Consequently, a re-conceptualization of AjD in the ICD-11 was achieved [4]. It is now being recognized as a full-threshold disorder with the core symptoms of preoccupation with the stressor (e.g., constant rumination) and failure to adapt (e.g., concentration problems) [14].

The importance of this change becomes apparent when looking at the current AjD health care situation. A large proportion of diagnosed individuals do not receive treatment at all [15]. The majority that do, receive it in primary health care settings. Here, AjD is just as likely to be treated with pharmacotherapy like other severe mental disorders; the crucial difference being, that there is no evidence base for this practice with AjD [4,16]. As Strain and Friedman recommend, the first-line of treatment for AjD is psychotherapy, where different general approaches like Cognitive Behavioral Therapy, Eye-Movement Desensitization and Reprocessing, Client-centered Psychotherapy and various brief psychological interventions have been tested with varying success [17,18,19,20]. Disorder-specific approaches are rare, but they do exist.

Regarding this scarcity of disorder-specific AjD treatment approaches, there have been various new impulses in the context of Internet-based interventions for AjD in recent years. One of the first digital AjD-specific treatments used Virtual Reality elements to enhance a face-to-face intervention. So-called “EMMA’s World”, proved to be an effective alternative to traditional treatment and led to significant improvements in areas of depression, relaxation and social interactions for participants [21]. A more recent development of the same group, called TAO, is currently being tested [22]. It combines approaches from cognitive-behavioral therapy (CBT) and Positive Psychology for the guided treatment of AjD. Preliminary findings show good results for both user-friendliness and acceptance for participants [23]. A different approach is pursued with BADI (Brief Adjustment Disorder Intervention), a self-guided intervention for AjD [24]. It combines exercises in relaxation, time management, mindfulness and coping with interpersonal difficulties. Results of a randomized controlled trial show a medium effect size of the intervention on adjustment disorder symptoms [24]. Lastly, there is a new guided online intervention for chronic stress, that explicitly includes AjD. It uses a larger catalog of contemporary CBT techniques, e.g., exposure, sleep management and behavioral activation. A first trial showed moderate to large improvements in terms of perceived stress, as well as in functional impairment and work ability [24].

From a client’s point of view, internet-based interventions offer significant advantages, some of which make AjD uniquely suited for this approach [25,26]. First, there is the potential for optimal timing of the intervention. In principle, online interventions could be made available immediately after the occurrence of an adverse life event, for example, via smartphone. This also eliminates the stigma, that would typically be associated with both a strong reaction to a stressor and a visit of a therapist, and usually prevents many individuals from utilizing available help [27]. Finally, the main advantage of self-guided interventions, in particular, is their enormous scalability. It allows not just for consistent availability of services but also ensures economic viability. Both the cost of distribution and the marginal costs added by each new user, converge towards zero with larger numbers of participants.

At the same time, digitalization also opens new possibilities for researchers and practitioners. A central idea is an iterative and incremental approach, as it is part for example, of the agile software development framework [28]. Instead of strictly sequential production, one assumes continuous development of an intervention, driven by constant learning. This has various advantages. Firstly, it allows for rapid iterations of just specific parts of either the content or technology. Scope and functionality can be efficiently expanded based on momentary resources and needs [26]. Secondly, the software allows for detailed analytics of use. With little additional effort, different functionalities or variants of modules can be tested in direct comparison [29]. Thirdly, the translational lag with the transfer of current research findings into clinical practice is significantly shorter than with traditional approaches [30]. Altogether, this bears the potential to significantly improve the quality and availability of care for individuals in need on a long-term basis.

These considerations form the basis of this study and its two objectives: Firstly, the development of a sustainable self-guided intervention for AjD called “Back to your own life (German acronym: ZIEL).” Secondly, to test the efficacy of this intervention, compared to a care as usual control group (CAU). We hypothesized that the active treatment condition would be superior to CAU on measures of AjD symptom severity and that effects would be stable in the three-month follow-up. The goal is to contribute and to expand the growing efforts in the field of AjD research and care.

## 2. Experimental Section

### 2.1. Study Design

This randomized controlled trial (RCT) compared an immediate intervention group with a CAU-only control group. The program was only accessible during the intervention stage. The participants of the control group got their access after the four-week post-assessment. The immediate intervention group was followed up until three months after randomization to examine the stability of potential gains. The trial was registered with Clinicaltrials.gov and was approved by the Ethics Committee of the School of Arts and Science at the University of Zürich, Switzerland (1 February 2016) [31].

### 2.2. Recruitment

We recruited individuals from the general population from January 2018 until March 2019. For this purpose, we used a study recruitment web page, which was advertised on various websites, forums, and social media. Additionally, we advertised the study at various psychiatric hospitals around Switzerland. The study web page presented general information about adjustment problems and AjD, an outline of the study, a link to 24 h emergency phone numbers, and a registration form.

Individuals who registered received detailed information about the study via e-mail. Individuals who signed informed consent were asked to complete online self-report questionnaires. Based on the answers, the eligibility criteria were assessed. For inclusion, the following criteria had to be met: Minimum age of 18 years, the existence of an emergency address and a life event between two weeks and two years before the participation that still negatively impacts their lives. The latter recorded by means of the Adjustment Disorder-New Module 20 (ADNM-20) stressor list.

The following criteria led to exclusion: Moderate or severe depressive symptoms (BDI > 18), suicidality (BDI suicidality item > 1), a diagnosis of psychotic, bipolar or other serious mental or physical disorders requiring immediate treatment. Individuals who did not meet the criteria were referred to adequate services and had the opportunity to access the intervention outside of the study.

### 2.3. Enrollment

**Figure 1:**
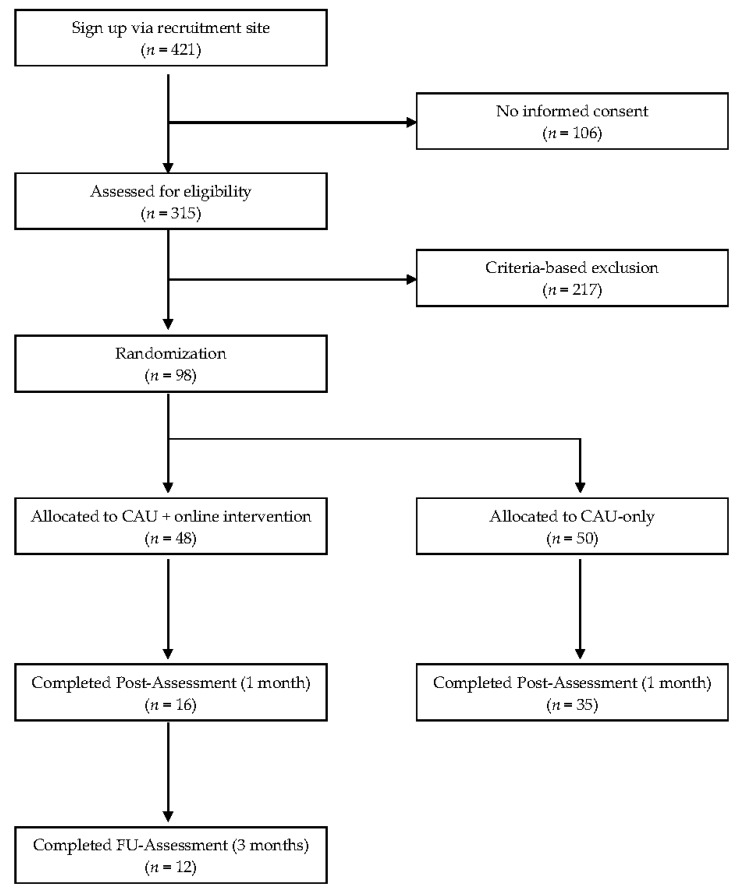
Participant flow.

A flowchart of the enrollment sequence is depicted in Figure 1. A total of 421 individuals signed up on the recruitment site, 315 gave informed consent and completed the baseline questionnaires. Out of those, 217 had to be excluded based on the inclusion and exclusion criteria. The remaining 98 participants were randomly assigned to one of the two trial conditions: the CAU plus internet intervention condition or the CAU control condition. Randomization was carried out by an independent researcher at the University of Bern. The independent researcher used anonymized numbers for the allocation, via a pre-produced 1:1 ratio, random sequence [32]. The allocation list was concealed from the investigators and participants. After the randomization, the participants received an email regarding their allocation.

### 2.4. Online Intervention

#### 2.4.1. Platform

Technologically, we wanted to create the conditions for continuous in-house intervention-development. A platform should enable us to efficiently adapt to advancements in research and on the end-user side. Consequently, we have aimed to implement powerful open source components whenever possible. One example would be the front-end toolkit Bootstrap that we used for mobile-first interface design [33]. In addition to high quality, the use of popular tools provides good accessibility for potential follow-up projects.

Another aim that guided development was a good user experience (UX) throughout the study. One instance of how this goal manifests itself, is the so called reduction of friction. In the context of UX, this is understood to mean that the intervention is used with as little disruption and frustration as possible to prevent users from abandoning their tasks at hand. An example of this would be the registration, as the first entry point to the study. Contrary to a common multi-step procedure, ZIEL-users could enter the registration-process with just one click of a button.

#### 2.4.2. Intervention

The intervention is based on a manual by Bachem and Maercker [34]. This manual is aimed at AjD for burglary victims and has already been successfully tested in a paper-based version [35]. Based on the theoretical model of AjD for the ICD-11, it integrates evidence-based techniques from the areas of post-traumatic stress disorder, anxiety disorders and depression [36]. The intervention is to be carried out over 4 weeks, whereby the content can be freely chosen by the users according to their current needs or symptoms.

In the first part, users are introduced to the concept of AjD and guided to assess their current symptom burden. Building on this, they get support in deciding whether the intervention is appropriate or whether it is better to make use of traditional support services. This is followed by a second part with self-help exercises, modelled along symptoms or symptom clusters as typically experienced by those affected.

In the first section on sense of self, the stress response and previous coping strategies are examined in more detail, also in light of existing risk and protective factors. In the second section on coping, a series of cognitive strategies are introduced in order to learn how to deal better with presenting burdens. These include techniques such as stopping thoughts from ruminating, as well as correcting cognitive biases (i.e., addressing preoccupations). In the third section on activation, the user deals primarily with the utilization of personal resources at various levels. This includes functional goal setting as well as e.g., the initiation of physical activities (i.e., addressing failure to adapt symptoms). Lastly, the section on recovery covers how activity and rest phases can best be kept in balance in the future, as well as a number of relaxation techniques. Table 1 depicts the details of the content sections.

**Figure A1:**
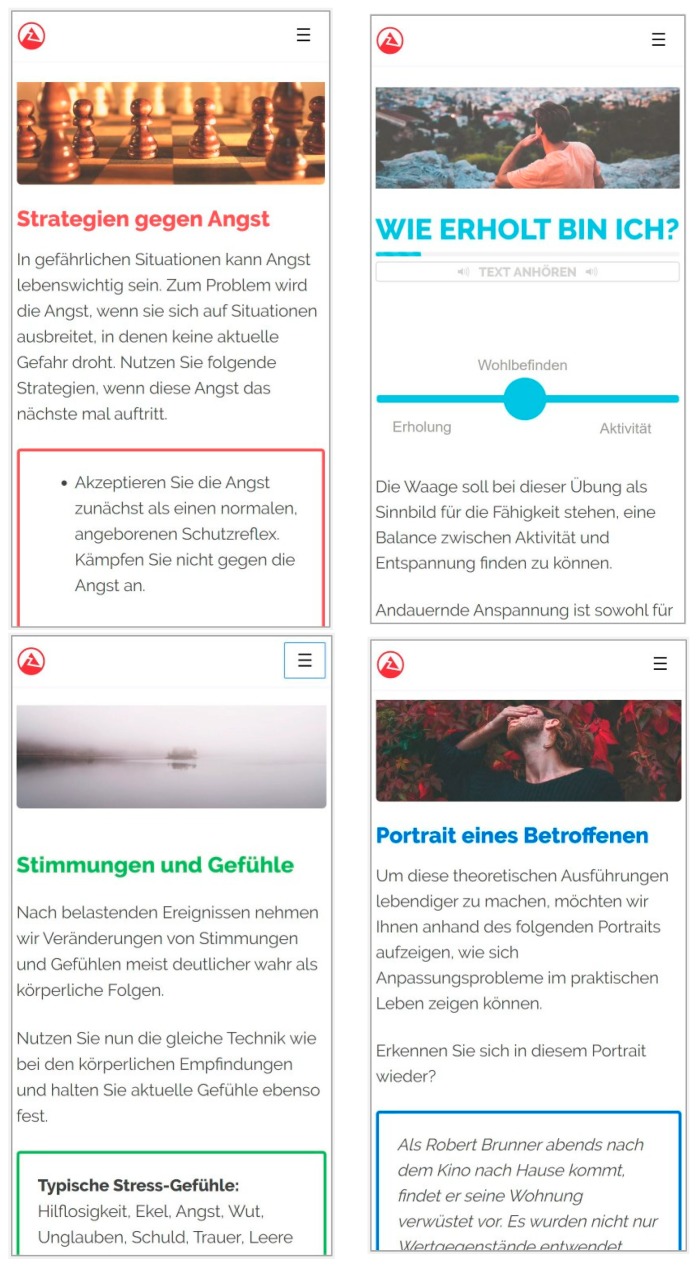
Illustrative Screenshots of the ZIEL Intervention.

The preservation of a self-directed approach resulted from the perspective of a possible scalable implementation in a standard care setting. After a negative life event, there is a time window of about one month in which a possible development of AjD takes place [14]. In order to support affected people to a relevant extent on such short notice, a reliance on skilled workforce would not be feasible regarding availability and organization. Closely related is the decision in favor of a completely anonymous and automated usage scenario. Social barriers to timely care should be eliminated as far as possible. Finally, the content was re-written agnostic of a specific stressor, restructured into shorter text-sections and additionally made available as audio versions. Figure A1 shows illustrative screenshots of the intervention.

### 2.5. Outcome Measures

#### 2.5.1. Adjustment Disorder Symptom Severity

The Adjustment Disorder-New Module 20 (ADNM-20) is a self-report questionnaire to track life events and identify adjustment issues. In the first part, it records acute and chronic life events by means of a semi-structured stressor list. In the second part, the AjD core symptoms of preoccupation and failure to adapt (4 items each) as well as accessory symptoms of avoidance, depressive mood, anxiety and impulse disturbance (12 items) are measured. All 20 items are measured on a 4-point Likert-type scale (1 = *never*, 4 = *often*) [37]. Additionally, a total sum score indicates the overall symptom burden and allows for the identification of high risk for AjD (score above 47.5) [38]. The scale offers satisfactory psychometric properties as shown in previous studies [36,37]. The internal consistency of the ADNM-20 in the present study was good for the sum score (Cronbach’s α 0.85), and acceptable for the subscales preoccupations (Cronbach’s α 0.78), failure to adapt (Cronbach’s α 0.73) and good for the accessory symptoms (Cronbach’s α 0.83).

#### 2.5.2. General Psychopathology

The Brief Symptom Inventory, Short Form (BSI-18) is a self-report questionnaire to assess general psychological distress. Syndromes of somatization, depression, and anxiety are measured by 18 items on a 5-point Likert-type scale (0 = not at all, 4 = very strong) [39]. The BSI-18 exhibits robust psychometric qualities in previous studies [38]. In the present study, Cronbach’s α was 0.82.

#### 2.5.3. Depressive Symptoms

The Beck Depression Inventory (BDI) is a self-report questionnaire to assess depressive symptoms. Each of the 21 items is rated on a 4-point Likert-type scale (0 = not at all, 3 = very strong) [40]. The BDI offers sufficient psychometrics properties [41]. In the present study, Cronbach’s α was 0.77.

#### 2.5.4. Quality of Life

The Short Form Health Survey–12 (SF-12) is a self-report questionnaire to assess health-related quality of life. Both a physical and a mental health index are measured by 12 items on a 5-point Likert-type scale [42]. The instrument shows robust psychometrics properties [43]. In the present study, Cronbach’s α for the mental health subscale was 0.78 and for the physical health subscale, 0.76.

#### 2.5.5. Expectations about Treatment

The Credibility/Expectancy Questionnaire (CEQ) is a self-report questionnaire to assess treatment expectancy and the credibility of its rationale. The subscales of treatment credibility and outcome expectation are measured by six items in total, each on a scale of 1 (not at all) to 9 (very much). The CEQ exhibits robust psychometric qualities [44]. In the present study, Cronbach’s α was 0.88.

#### 2.5.6. Usability

The System Usability Scale (SUS) is a self-report questionnaire to assess the usability of a system. Each of the ten items was adapted to the use-case and is measured on a 5-point Likert-type scale (1 = strongly agree, five strongly disagree) [45]. The SUS offers robust psychometric properties [46]. In the present study, Cronbach’s α was 0.84.

#### 2.5.7. Adherence

The intervention platform automatically registered various indices of adherence for each anonymized account-ID: Number of logins, individual pageviews, pageviews per login, total and average time spent in the intervention.

### 2.6. Power Analysis

The power analysis was conducted with G*Power 3 to determine the appropriate sample size for the detection of differences between the two groups [47]. We aimed at the detection of a medium effect size of 0.5, based on previous research by Eimontas et al. [22]. Accordingly, a power analysis showed that with an alpha error level of 0.05 and a power of (1-beta) of 0.80 about 128 individuals would be needed.

### 2.7. Statistical Analysis

To test group differences in both demographic data and baseline measures, independent sample *t*-tests, respectively *χ*^2^-tests for nominal data variables were used. Differential outcomes at posttreatment were evaluated according to an intention-to-treat principle using a mixed-model repeated-measures analysis of variance with time (pre-post) as a within-group factor and treatment condition as a between-group factor. This approach was favored as it uses all available data of each subject. Missing values are not substituted; rather parameters of missing values are estimated [48]. Within- and between-group effect sizes (Cohen’s d) were calculated based on estimated means and the pooled standard deviation from the observed means. Within-group changes in outcome scores from posttreatment to follow-up were analyzed using paired *t*-tests for people who completed the post and the follow-up-assessment in the intervention group only. To test predictions to the outcome, we calculated linear regression models regressing each adherence measure on the 4-week primary outcome (ADNM-20) controlling for baseline scores in the intervention group. Post hoc tests were Bonferroni corrected for multiple comparisons. All analysis was performed in R and the package lme4 [49,50].

## 3. Results

### 3.1. Pre-Treatment Evaluation

The conditions in both groups did not differ in terms of AjD symptom burden or demographic characteristics. The incidence of AjD in the intervention group was 71% (*n* = 34) and 72% in the CAU group (*n* = 36). Table 2 provides corresponding details for demographic characteristics. Likewise, the perception of credibility and expectancy of the intervention was the same for participants in both conditions (*p* > 0.47).

### 3.2. Dropout Analysis

Overall, 47 (active, *n* = 32; CAU, *n* = 15) participants (48%) did not complete the posttreatment assessment, even though they had been invited three times in weekly intervals via automated email. The difference in response between the two groups is significant (*p* < 0.01). This can likely be attributed to the fact, that the control group did not gain access to the intervention until after completing the post-assessments. Reasons for dropping out remained unknown since there was no way to reach and question the respective users. As for predictors of dropout, there were no significant differences observed in terms of demographics, pre-treatment or post-treatment scores (all *p*s > 0.21) between those who provided data and those who did not.

### 3.3. Treatment Outcomes

The observed and estimated means for the self-report questionnaires are presented in Table 3. Mixed-model linear regression analysis with group as a fixed factor and time as a repeated factor (pre-post) were conducted for each of the dependent outcome measures.

For the primary outcome, the effect of the ADNM-20 was not qualified by significant Group × Time interactions for either the sum score (F_1,96_ = 2.38, *p* = ns), or for the subscales (F_1,96_ = 0.03–3.13, *p* = ns; see Table 3), meaning that the symptom severity did not decrease significantly in the intervention group compared to the control group. Between-group effect sizes based on estimated means, corrected for baseline differences, were small for the sum score (*d* = 0.31) and small to medium sized for the subscales (*d* = 0.03–0.51; see Table 3). Within-group comparisons based on estimated means in the treatment group showed large effect sizes for the sum score (*d* = 1.04) and small to medium effect sizes for the subscales (*d* = 0.27–0.74; see Table 3). Within-group effect sizes in the control group were medium-sized for the sum score (*d* = 0.70) and small to medium-sized as well for the subscales (*d* = 0.37–0.69; see Table 3).

As for secondary outcomes, the effect of the BDI showed significant Group × Time interactions (BDI: F_1,96_ = 19.5 *p* < 0.01). At post, between-group effect sizes based on estimated means were *d* = 0.89, meaning that for the intervention group, there was a large effect in terms of depressive symptom decrease compared to the control group. Within-group comparisons based on the estimated means in the treatment group showed large effect sizes (BDI: *d* = 0.95). In contrast, within-group effect sizes in the control group were negligible (BDI: *d* = 0.20). Treatment effects for the intervention group at three-month-follow-up were stable (pre–follow-up, *d* = 0.95) as no significant differences could be detected in comparison to the effects at post (*p* > 0.30).

For the SF-12 mental health subscale, there was a significant Group × Time interaction (SF-12_MH_: F_1,96_ = 13.52, *p* < 0.01). At post, between-group effect sizes based on estimated means were *d* = 0.74, meaning that for the intervention group, there was a medium effect in terms of increased mental health-related quality of life compared to the control group at post. Within-group comparisons based on the estimated means in the treatment group showed small effect sized at post (SF-12_MH_post_: *d* = 0.31) and significantly improved to medium effect size at follow-up (SF-12_MH_fu_: *d* = 0.68, *p* < 0.01). In contrast, within-group effect sizes in the control group were negligible at post (SF-12_MH_: *d* = 0.02). For the SF-12 physical health subscale, there were no significant Group × Time interactions detected (SF-12_PH_: F_1,96_ = 3.16, *p* = 0.08).

### 3.4. Diagnostic Status Pre- and Post-Treatment

In total, 51 participants filled out the post-treatment questionnaire (Active, *n* = 16 (33%), CAU, *n* = 35 (70%)). According to the self-report at post-treatment, 15 out of 48 participants in the intervention group (31%) did not meet the criteria for AjD. In contrast, 14 participants in the control group (28%) could be considered remitted after four weeks.

### 3.5. Program Usage and Usability

The intensity in which the intervention was used shows a high degree of heterogeneity within the user group. The average time spent in the intervention (total) was 43 min (SD = 106.6), while the average duration of a session was 8 min (SD = 11). The average number of sessions (total) was 2.7 (SD = 5.2). The usability of the intervention was rated as above average by the users. The average SUS score was 73 (SD = 18), which translates to an adjective rating of “good” [51].

### 3.6. Predictors of Outcome

To investigate potential predictors of outcome, we used regression analyses, predicting 4-week primary and secondary outcomes, controlled for baseline scores. For these analyses, we only used data of participants that logged in at least once and completed the post-treatment questionnaires. None of the pre-treatment variables such as age, sex, marital status, occupational situation, education or psychotherapeutic treatment had any significant relation to the primary outcome (all *ps* > 0.24). At the same time, none of the indicators of program usage such as number of sessions or time spent in the intervention were significantly associated with the treatment outcomes for both groups of participants (all *p*s > 0.27).

## 4. Discussion

The present research aimed to develop a self-guided intervention for adjustment problems and to test its efficacy compared to a control group. Contrary to our hypothesis, there were no significant differences between the intervention group and the control group regarding the primary outcome measure ADNM-20 and its subscales. Both groups showed significantly lower AjD symptom burden from baseline to post-treatment. This result and the within-group effect size of *d* = 1.04 in the treatment group are in line with the results obtained with the original manual by Bachem and Maercker, what indicates its adequate online implementation [32,33]. The between-group effect size of *d* = 0.31 was not significant, whereas, the study was underpowered to detect small effects like this.

Although both groups showed similar improvements for the primary outcome, the intervention group showed a significant between-group effect on the reduction of depressive symptoms (*d* = 0.89). Effects within the intervention group could also be maintained over the three-month follow-up period. This is an encouraging result, which could not yet be shown in previous studies on internet interventions for AjD [22].

Likewise, there was a significant between-group effect regarding the improvement of mental health-related quality of life at post (*d* = 0.74). Within-group comparisons in the intervention group showed significant improvements over all three points of measurement, respectively (SF-12_MH_post_: *d* = 0.31, SF-12_MH_fu_: *d* = 0.68). For the physically related quality of life, there were no significant effects detected (SF-12_PH_: *d* = 0.33, *p* = 0.08), which again could be related to the lack of power to detect small effect sizes in the present study.

The overall results are further consistent with those of the BADI self-guided approach to AjD treatment by Eimontas et al. [22]. It showed medium effect sizes regarding the AjD symptom reduction, starting from a comparable severity of symptom burden in the pre-treatment evaluation. In contrast to the present study, however, the control group showed no comparable improvements.

In a wider context, the results of the present study stand in accordance with previous research that supports the viability of self-guided approaches in different contexts [52,53]. We were able to show that highly scalable self-guided interventions have a positive effect on users. However, the effects are still small and many dropouts can be expected.

### 4.1. Limitations

Several limitations to this study need to be acknowledged. First, the sample consisted of self-selected participants. It can be assumed that already a positive opinion regarding internet interventions prevailed. This restricts generalizability, but on the flipside, also reflects a realistic health care scenario. Second, the study suffers from a high dropout rate of 48% (pre-post). Third, the results of the study are solely based on self-report measures, which however allows for scalability of the approach. Fourth, we had a relatively heterogeneous sample of participants regarding symptom burden. Fifth, unexpectedly, the organizational- and time-restrictions of the project did not allow the recruitment efforts to be extended in such a way as to achieve the number of participants required for the assumed effects. This calls for changes in future studies and restricts the generalizability of the current results, demanding their appropriately cautious interpretation. Lastly, from an ethical perspective, we deemed it important to not extend the delay for the waiting list further than needed in the potential period of onset of AjD as a transient disorder. However, this not only made a comparison between experimental and control group at three months impossible, but also clearly limits the informative value on account of the temporal stability of effect beyond this period.

### 4.2. Implications and Learnings

Even though it is an empirically common phenomenon, especially for self-guided interventions, the low adherence to the intervention, respectively high dropout of the participants, should not be overlooked. Several studies have already demonstrated meaningful relation to treatment outcome [22,54]. Thus, it is a positive sign, that significant effects have been found for ZIEL, despite the relatively small “adherence-dosage”. However, a solution-oriented approach to the problem is mandatory and represents an important aspect of the further development of the intervention. We have to assume that we were not able to match the needs of users, e.g., in terms of technical or content-related aspects [55].

To improve adherence, we see different steps that could be implemented in the next iteration [56]. First, the integration of automated reminders should be able to increase engagement [57]. Complementary to this, the addition of automatic feedback on tasks and virtual rewards for favorable actions could be a valuable investment that we want to pursue. On a more general level, we see the need to improve the product-market-fit of the intervention in the broadest sense. To better be able to match user needs, we plan to focus and collaborate with specific target groups, based on classes of AjD-relevant life events like e.g., divorce. This would make it possible to generate greater personal relevance for the user on the one hand. On the other hand, we could target specific needs more directly and learn faster from focused user feedback. An example of such an approach in a guided format is the internet-based intervention for adaption problems after separation or divorce, called LIVIA, which showed significant improvements and moderate effect sizes for participants [58].

A positive and fruitful experience which we gained during the work on the present study, was with the closed loop between the development of both the content and the software. While there is currently a trend in our field towards outsourcing the technical implementation, it has proven very efficient for us to understand the content and IT as two sides of the same coin. Based on an agile strategy, one can make early decisive decisions for later developments. In other words, the hope is that initial investment in interdisciplinary teams will pay off several times over, in the lifecycle of fruitful projects. The approach becomes even more relevant in an academic context, with a multitude of parallel projects, changing financing situations and continuously changing teams.

In conclusion, the present study supports the current positive findings of efficient internet-based treatment of AjD. It expands the current state of knowledge with an extension of possible approaches to treatment and conceptual considerations regarding the process of realization. Given the potential reach and impact of a low threshold, high scalability intervention for AjD, sustainable research efforts are required.

## Figures and Tables

**Table 1:** Section overview.

Section 1: Introduction	Psychoeducation; learning to evaluate the psychological burden caused by a life event; deciding about the adequacy of the intervention.
Section 2: Sense of self	Analyzing the individual adjustment reaction; learning about risk and protective factors; inspecting individual former coping strategies.
Section 3: Coping	Recognizing and correcting cognitive biases; writing a narrative exposure exercise; managing anxiety and rumination.
Section 4: Activation	Physical exercising; setting realistic positive goals; regaining positive experiences; evaluating milestones of the personal history.
Section 5: Recovery	Learning to balance activity and relaxation; getting to know various approaches to relaxation; improving sleep and imaginative techniques.

**Table 2:** Pre-Treatment Evaluation.

	Intervention Group *n* = 48	CAU-Only Group *n* = 50	Statistic
Mean age, years (SD)	40.54 (13.0)	40.28 (12.8)	*t*(96) = 0.10 *p* = 0.92
Gender *n* (%)			
Female	40 (83.3)	44 (88.0)	Chi^2^ = 0.44 *p* = 0.51
Male	8 (16.7)	6 (12.0)	
Marital status, *n* (%)			
Single	11 (22.9)	10 (20.0)	Chi^2^ = 2.06 *p* = 0.72
Married	28 (37.5)	24 (48.0)	
Living together	10 (20.8)	11 (22.0)	
Divorced	7 (14.6)	4 (8.0)	
Widowed	2 (4.2)	1 (2.0)	
Number of kids, *n* (%)			
None	23 (47.9)	27 (54.0)	Chi^2^ = 3.36 *p* = 0.50
One	7 (14.6)	11 (22.0)	
Two	13 (27.1)	10 (20.0)	
Three	4 (8.3)	1 (2.0)	
More than three	1 (2.1)	1 (2.0)	
Employment, *n* (%)			
Full-time paid work	13 (27.1)	23 (46.0)	Chi^2^ = 5.60 *p* = 0.35
Part-time paid work	19 (39.6)	15 (30.0)	
Unemployed	2 (4.2)	2 (4.0)	
At home parent	4 (8.3)	1 (2.0)	
Student	5 (10.4)	3 (6.0)	
Retired	5 (10.4)	6 (12.0)	
Highest education, *n* (%)			
Compulsory school	1 (2.1)	0 (0.0)	Chi^2^ = 5.06 *p* = 0.17
Apprenticeship	15 (31.3)	8 (16.0)	
College	5 (10.4)	4 (8.0)	
University	27 (56.3)	38 (76.0)	
Current psychological treatment, *n* (%)			
Yes	15 (31.3)	9 (18.0)	Chi^2^ = 2.33 *p* = 0.13
No	33 (68.8)	41 (82.0)	
Current medications, *n* (%)			
Yes	5 (10.4)	2 (4.0)	Chi^2^ = 1.52 *p* = 0.22
No	43 (89.6)	43 (86.0)	
Experience with E-Mental Health, *n* (%)			
Yes, good	5 (10.4)	4 (8.0)	Chi^2^ = 0.17 *p* = 0.68
Yes, bad	0 (0.0)	0 (0.0)	
No	43 (89.6)	46 (92.0)	

**Table 3:** Treatment Outcomes. Observed and Estimated Means for Primary and Secondary Outcome Measures and Within- and Between-Group effect-sizes.

	Pre-Treatment	Post-Treatment (Observed)		Post-Treatment (Estimated)		Follow-Up (Observed)		Post-Treatment Between-Group Comparisons (Group by Time)	Pre-Post Within-Group Effect Sizes (Estimated, ** for *p* ≤ 0.01)	Between-Group Effect Sizes at Post (Est.)	
Measure	*n*	M (SD)	*n*	M (SD)	*n*	M (SE)	*n*	M (SD)	F and df	Cohen’s *d* (95% CI)	Cohen’s *d* (95% CI)
ADNM-20 Preoccupation											
CAU	50	13.12 (1.79)	35	11.41 (1.99)	50	11.70 (2.01)			F_1,96_ = 0.03	0.69 (0.47–0.91)	0.03 (−0.43–0.50)
Intervention	48	12.23 (2.22)	16	11.50 (2.15)	48	11.63 (2.27)	12	10.17 (2.37)		0.27 (0.09–0.45)	
ADNM-20 Failure to adapt											
CAU	50	9.85 (2.73)	35	8.68 (2.30)	50	8.66 (2.29)			F_1,96_ = 3.13	0.37 (0.18–0.57)	0.40 (−0.07–0.87)
Intervention	48	9.35 (2.12)	16	7.75 (2.25)	48	7.88 (2.09)	12	7.92 (2.07)		0.74 (0.52–0.93)	
ADNM-20 Accessory symptoms											
CAU	50	31.42 (5.70)	35	28.05 (4.82)	50	28.32 (5.41)			F_1,96_ = 0.33	0.55 (0.36–0.75)	0.51 (0.02–1.00)
Intervention	48	28.00 (6.37)	16	25.32 (6.64)	48	25.35 (6.38)	12	22.75 (5.85)		0.41 (0.18–0.63)	
BDI											
CAU	50	11.78 (3.87)	35	9.86 (4.44)	50	11.20 (4.78)			F_1,96_ = 19.52 **	0.13 (−0.06–0.31)	0.83 (0.41–1.25)
Intervention	48	10.83 (5.13)	16	9.76 (6.72)	48	10.23 (4.80)	12	4.44 (4.90)		0.72 (0.48–0.97)	
BSI-18											
CAU	50	12.52 (8.87)	35	11.41 (8.67)	50	10.80 (7.72)			F_1,96_ = 0.49	0.20 (0.02–0.39)	0.17 (−0.24–0.57)
Intervention	48	14.56 (9.18)	16	11.08 (6.22)	48	12.17 (8.72)	12	5.27 (5.46)		0.27 (0.14–0.39)	
SF-12_PH_											
CAU	50	14.62 (3.34)	35	14.79 (3.29)	50	14.52 (3.15)			F_1,96_ = 3.16	−0.03 (−0.22–0.15)	0.36 (−0.04–0.76)
Intervention	48	14.60 (3.09)	16	15.20 (3.12)	48	15.60 (2.88)	12	18.00 (2.68)		0.33 (0.09-0.58)	
SF-12_MH_											
CAU	50	15.96 (2.58)	35	15.68 (2.25)	50	15.96 (2.47)			F_1,96_ = 13.52 **	0.02 (−0.24–0.27)	0.74 (0.32–1.16)
Intervention	48	16.98 (2.62)	16	17.73 (2.40)	48	17.77 (2.40)	12	18.36 (2.01)		0.31 (0.07–0.55)
